# Longitudinal lipidomic profiles of left ventricular mass and left ventricular hypertrophy in American Indians

**DOI:** 10.1172/jci.insight.181172

**Published:** 2024-10-15

**Authors:** Mingjing Chen, Zhijie Huang, Guanhong Miao, Jin Ren, Jinling Liu, Mary J. Roman, Richard B. Devereux, Richard R. Fabsitz, Ying Zhang, Jason G. Umans, Shelley A. Cole, Tanika N. Kelly, Oliver Fiehn, Jinying Zhao

**Affiliations:** 1 Department of Epidemiology, College of Public Health & Health Professions and College of Medicine, University of Florida, Gainesville, Florida, USA.; 2 Department of Epidemiology, Tulane University School of Public Health and Tropical Medicine, New Orleans, Louisiana, USA.; 3 Division of Cardiology, Weill Cornell Medical College, New York, New York, USA.; 4 Missouri Breaks Industries Research Inc., Eagle Butte, South Dakota, USA.; 5 Department of Biostatistics and Epidemiology, University of Oklahoma Health Sciences Center, Oklahoma City, Oklahoma, USA.; 6 MedStar Health Research Institute, Hyattsville, Maryland, USA.; 7 Georgetown-Howard Universities Center for Clinical and Translational Science, Washington DC, USA.; 8 Texas Biomedical Research Institute, San Antonio, Texas, USA.; 9 Department of Medicine, University of Illinois Chicago, Chicago, Illinois, USA.; 10 West Coast Metabolomics Center, UCD, Davis, California, USA.

**Keywords:** Cardiology, Metabolism, Cardiovascular disease, Lipoproteins

## Abstract

**BACKGROUND:**

Left ventricular hypertrophy (LVH) and dyslipidemia are strong, independent predictors for cardiovascular disease, but their relationship is less well studied. A longitudinal lipidomic profiling of left ventricular mass (LVM) and LVH is still lacking.

**METHODS:**

Using liquid chromatography–mass spectrometry (LC-MS), we repeatedly measured 1,542 lipids from 1,755 unique American Indians attending 2 exams (mean, 5 years apart). Cross-sectional associations of individual lipid species with LVM index (LVMI) were examined by generalized estimating equation (GEE), followed by replication in an independent biracial cohort (65% White, 35% Black). Baseline plasma lipids associated with LVH risk beyond traditional risk factors were identified by logistic GEE model in American Indians. Longitudinal associations between changes in lipids and changes in LVMI were examined by GEE, adjusting for baseline lipids, baseline LVMI, and covariates.

**RESULTS:**

Multiple lipid species were significantly associated with LVMI or the risk of LVH in American Indians. Some lipids were confirmed in Black and White individuals. Moreover, some LVH-related lipids were inversely associated with risk of coronary heart disease (CHD). Longitudinal changes in several lipid species were significantly associated with changes in LVMI.

**CONCLUSION:**

Altered fasting plasma lipidome and its longitudinal change over time were significantly associated with LVMI and risk for LVH in American Indians. Our results offer insight into the role of individual lipid species in LV remodeling and risk of LVH, independent of known risk factors.

**FUNDING:**

This study was supported by the NIH grant (R01DK107532). The Strong Heart Study has been funded in whole or in part with federal funds from the National Heart, Lung, and Blood Institute, NIH, Department of Health and Human Services, under contract nos. 75N92019D00027, 75N92019D00028, 75N92019D00029, and 75N92019D00030.

## Abstract 2

Exploring the association between dyslipidemia and heart chamber thickening, our study used lipidomics to uncover specific blood lipids that may predict heart disease risk.

## Graphical abstract

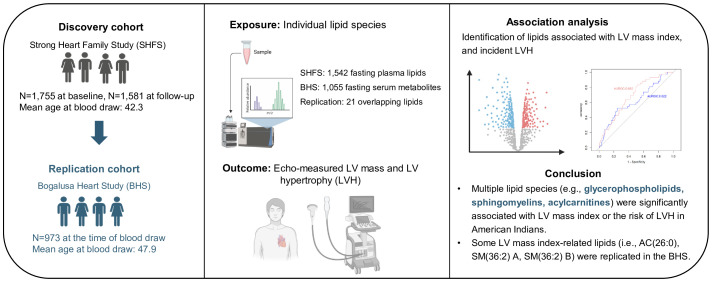

## Introduction

Dyslipidemia, defined as elevated total or low-density lipoprotein cholesterol (LDL-c) levels or as a low level of high-density lipoprotein cholesterol (HDL-c), is an established risk factor for cardiovascular disease (CVD) (1, 2). American Indians suffer from disproportionately high rates of CVD and its risk factors, such as obesity and diabetes (3, 4). Left ventricular hypertrophy (LVH), defined as an increase in the left ventricular mass (LVM) due to thickening of the left ventricular (LV) wall (5), has been shown to be an independent risk factor for CVD in various ethnic groups (6–9) including American Indians (10). Additionally, lipid dysregulation has been associated with LVM and risk of LVH (11–13). However, the mechanisms underlying the association between dyslipidemia, LVH, and CVD are not fully understood. The routinely used lipid panels in clinical practice measure the blood concentrations of bulk lipoproteins, but these methods do not distinguish the hundreds to thousands of individual lipid species in a blood sample. A comprehensive profiling of these individual lipid species (i.e., lipidome) is crucial to understanding the disease mechanism and identifying novel biomarkers for risk stratification, early prevention, or intervention of CVD.

Lipidomics is a mass spectrometry–based (MS-based) omics technology that can identify and quantify hundreds to thousands of individual lipid species in a biospecimen. Using this technology, previous studies have reported associations of altered lipid species, such as ceramides, triacylglycerols, sphingolipids, and phospholipids, with LVM and LVH in human populations (14, 15). However, these studies were largely cross-sectional and limited by small sample size and/or low coverage of the blood lipidome. To date, there have been limited large-scale lipidomic studies examining the association between individual lipid species and LV remodeling, especially in longitudinal settings. Here we report findings from what we believe to be the first large-scale longitudinal lipidomic profiling of LVM and LVH in 3,336 fasting plasma samples from 1,755 unique American Indians (1,755 at baseline; 1,581 at follow-up) attending 2 clinical exams (~5 years apart) in the Strong Heart Family Study (SHFS) (16). Our objectives include (a) identifying individual lipid species associated with LVM and incident LVH, beyond known risk factors; (b) examining whether LVH-related lipid species are associated with incident coronary heart disease (CHD) during an average 18-year follow-up; and (c) testing the longitudinal association between changes in plasma lipidome and changes in LVM over an average 5-year follow-up.

## Results

As shown in Table 1, the mean age of SHFS participants was 39.9 years at baseline and 45.1 years at follow-up. The mean LVM index (LVMI) was 38.7 (g/m^2.7^) and 39.4 (g/m^2.7^) at baseline and follow-up, respectively. Over an average 5 years of follow-up, 5.5% of participants developed incident LVH (all belong to eccentric hypertrophy). The mean age of Bogalusa Heart Study (BHS) (17, 18) participants was 47.9 years, and the mean LVMI was 38.6 (g/m^2.7^) at the time of blood drawn for metabolomic analysis.

### Plasma lipid species associated with LVMI.

At the SHFS baseline visit, we identified 289 lipid species (106 known) significantly associated with LVMI at *P* < 0.05. Of the 106 known lipids, 49 lipids remained statistically significant after multiple testing correction (*q* < 0.05). At the SHFS follow-up visit, 314 lipids (128 known) were associated with LVMI at *P* < 0.05. Of the 128 known lipids, 51 lipids remained statistically significant after multiple testing correction (*q* < 0.05). Meta-analysis combining results from both time points showed that 421 lipid species (152 known) were significantly associated with LVMI at *P* < 0.05. Specifically, 113 lipids — including 43 sphingomyelins (SMs), 40 phosphatidylcholines (PCs), 15 triacylglycerols (TAGs), 4 diacylglycerols (DAGs), 4 phosphatidylethanolamines (PEs), 3 cholesterol easters (CEs), 3 fatty acids (FAs), and cholesterol — were inversely associated, whereas 39 lipids — including 13 PCs, 6 PEs, 4 acylcarnitines (ACs), 4 SMs, 3 ceramides (CERs), 2 glycosylceramides (GlcCers), 2 phosphatidylinositols (PIs), 2 TAGs, CE(16:1), FA(22:1), and PG(17:0/19:0) — were positively associated with LVMI. Among them, 90 known lipids remained significant after multiple testing correction (*q* < 0.05).

**Figure 1:**
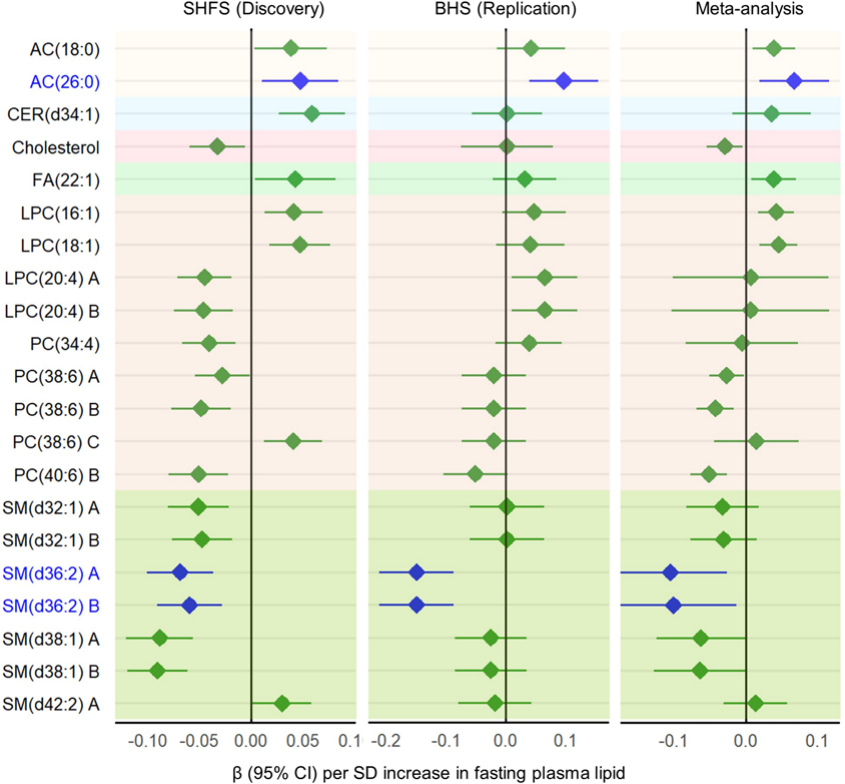
Plasma lipid species associated with left ventricular mass index (LVMI). Regression coefficients (βs) and 95% CIs in the Strong Heart Family Study (SHFS) were obtained by linear generalized estimating equation (GEE) models, adjusting for age, sex, study center, BMI, smoking, systolic blood pressure, diabetes, eGFR, LDL-c, and the use of lipid-lowering medication at the time blood sample was drawn. βs in the Bogalusa Heart Study (BHS) (replication) were obtained by linear regression model, adjusting for age, sex, race, smoking, systolic blood pressure, BMI, eGFR, diabetes, LDL, and the use of lipid-lowering medication. Only 21 known lipids with *P* < 0.05 in the SHFS that are also available in the BHS are shown. The names of lipids confirmed in the BHS (*q* < 0.05) are highlighted in blue. The letter A, B, or C in the name of lipids represents the appropriate isomer.

Of the 152 known lipids identified in the meta-analysis in the SHFS (*P* < 0.05), 21 lipids were also measured in the BHS (Supplemental Table 1; supplemental material available online with this article; https://doi.org/10.1172/jci.insight.181172DS1). Of these, 3 lipids [i.e., AC(26:0), LPC(20:4) A, LPC(20:4) B] were positively, whereas 2 lipids [i.e., SM(d36:2) A, SM(d36:2) B] were inversely associated with LVMI in the BHS at *P* < 0.05. Of these 5 lipids, 3 lipids [i.e., AC(26:0), SM(d36:2) A, SM(d36:2) B] remained significant after multiple testing correction (*q* < 0.05) with the same directions of association (Figure 1).

Of the 21 lipids measured in both cohorts, transethnic meta-analysis found that 5 lipids, including 2 ACs, 2 LPCs, and FA(22:1), were positively, whereas 3 lipids [i.e., PC(38:6) B, PC(40:6) B, SM(d36:2) A] were inversely associated with LVMI at *q* < 0.05.

### Baseline plasma lipid species predict incident LVH beyond known clinical factors.

**Figure 2:**
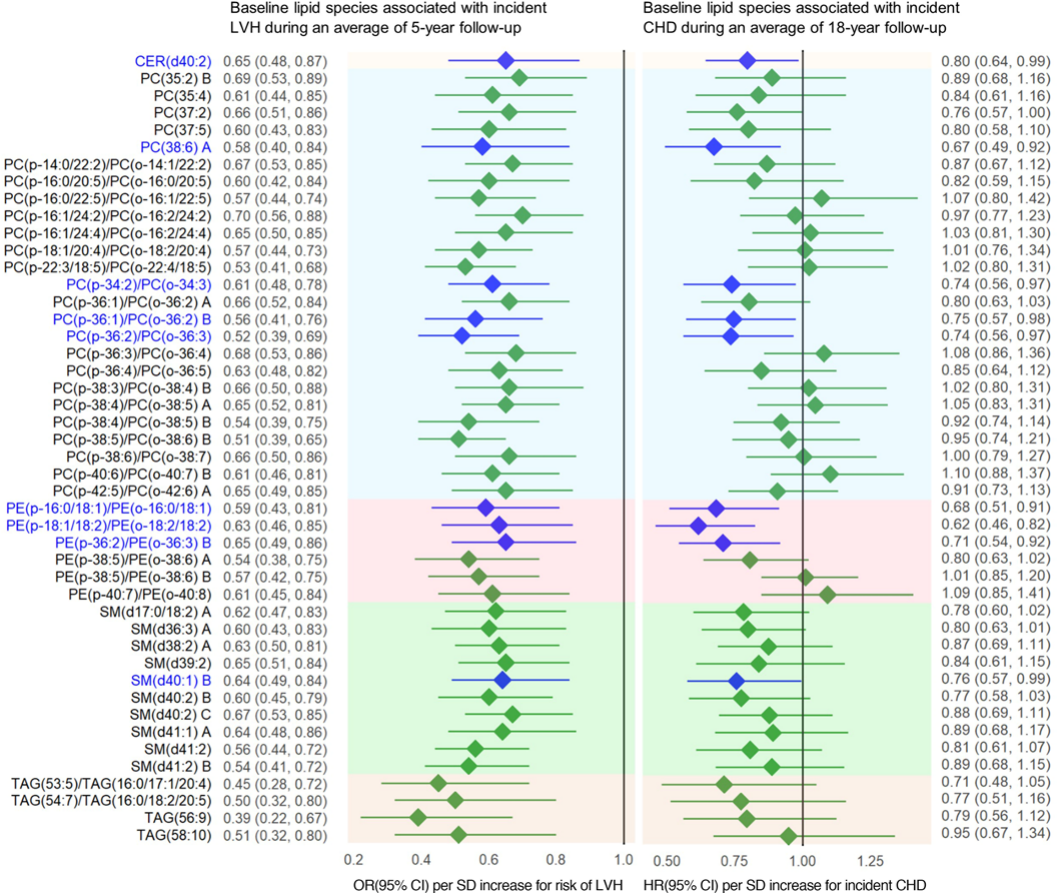
Baseline plasma lipids associated with risk of left ventricular hypertrophy (LVH) (mean follow-up: 5 years) in the SHFS. Several plasma lipid species (baseline) associated with incident LVH were also associated with incident coronary heart disease (CHD) (mean follow-up: 18 years). Odds ratios (ORs) were obtained by GEE, adjusting for age, sex, study center, BMI, smoking, systolic blood pressure, diabetes, eGFR, LDL-c, and the use of lipid-lowering medication at baseline. Hazard ratios (HRs) and 95% CIs were obtained by frailty Cox proportional hazards models, adjusting for same covariates plus HDL-c at baseline. Lipids with *P* < 0.05 are highlighted in blue. The letter A, B, or C in the name of lipids represents the appropriate isomers.

In the SHFS, we identified 310 lipids (134 known) significantly associated with incident LVH at *P* < 0.05. After correction for multiple testing, 46 lipids (of 134 known lipids) remained significant at *q* < 0.05 (Figure 2). Specifically, baseline levels of 46 lipids, including 25 PCs, 6 PEs, 10 SMs, 4 TAGs, and CER(d40:2), were inversely associated with the risk of LVH (odds ratios [ORs], 0.39–0.70).

**Figure 3:**
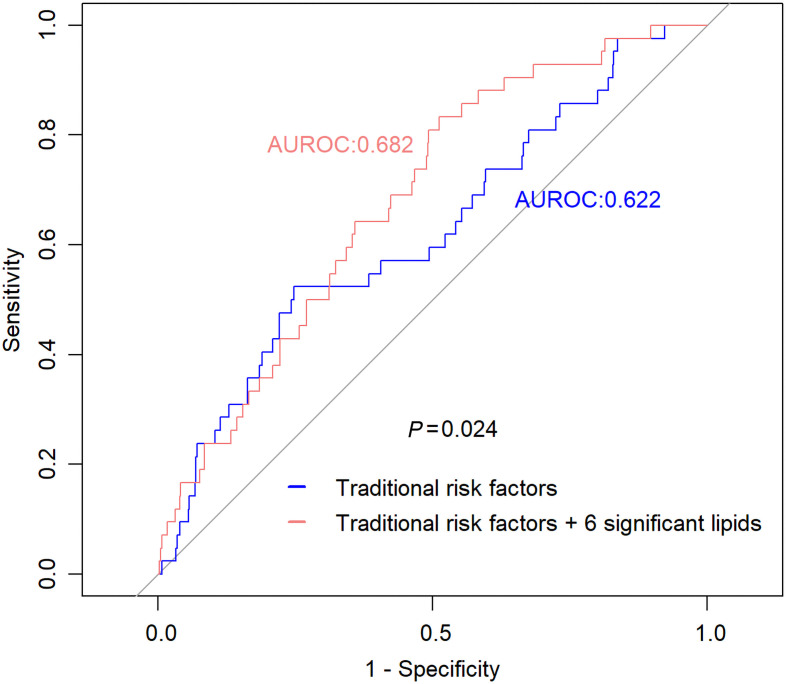
Incremental value of the identified plasma lipids in risk prediction for LVH. We used data from 2 study centers (North/South Dakota and Arizona) as training sample (*n* = 762, 42 cases), and those from another center (Oklahoma) (*n* = 675, 37 cases) as testing sample. The training sample was used for model training, and the testing sample was used to test classification performance. Model 1: traditional risk factors only, including age, sex, BMI, smoking, systolic blood pressure, diabetes, eGFR, LDL-c, and the use of lipid-lowering medication at baseline. Model 2: clinical factors + 6 significant lipids, including PC(p-16:0/22:5)/PC(o-16:1/22:5), PC(p-22:3/18:5)/PC(o-22:4/18:5), PC(p-34:2)/PC(o-34:3), PC(p-36:2)/PC(o-36:3), PC(p-38:5)/PC(o-38:6) B, and PE(p-38:5)/PE(o-38:6). Compared with the model that included clinical factors only (model 1), additional inclusion of plasma lipids (model 2) significantly increased risk prediction for LVH. AUROC increased from 0.622 to 0.682 (*P* = 0.024).

As shown in Figure 3, additional inclusion of the top 6 lipids (of 46 lipids), including PC(p-16:0/22:5)/PC(o-16:1/22:5), PC(p-22:3/18:5)/PC(o-22:4/18:5), PC(p-34:2)/PC(o-34:3), PC(p-36:2)/PC(o-36:3), PC(p-38:5)/PC(o-38:6) B, and PE(p-38:5)/PE(o-38:6) B, significantly improved risk prediction for LVH over clinical factors (AUROC increased from 0.622 to 0.682, *P* = 0.024).

### LVH-related lipids associated with incident CHD.

During an average of 18-year follow-up, 87 SHFS participants developed incident CHD. Of the 46 known lipids associated with incident LVH, higher baseline levels of 9 lipids [i.e., 4 PCs, 3 PEs, CER(d42:0), SM(d40:1) B] were also significantly associated with a lower risk of CHD (hazard ratios [HRs], 0.62–0.80), after adjusting for multiple testing (*q* < 0.05) and covariates including age, sex, study center, BMI, smoking, systolic blood pressure (SBP), diabetes, eGFR, LDL-c, HDL-c, and the use of lipid-lowering medication at baseline (Figure 2 and Supplemental Table 2).

### Longitudinal changes in lipid species associated with change in LVMI.

**Figure 4:**
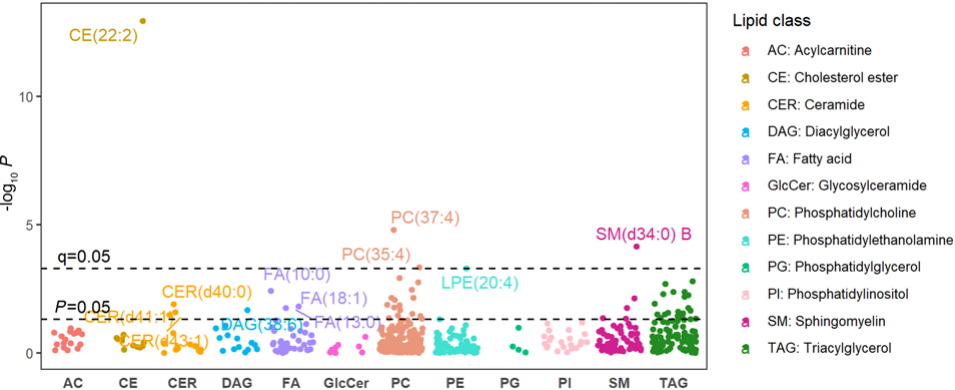
Manhattan plot displaying the longitudinal associations between change in plasma lipids and change in LVMI over an average of 5-year follow-up. The *x* axis, lipid classes; the *y* axis, –log_10_ *P*. Different colors represent different lipid categories. The dashed lines represent significance level at *P* = 0.05 and *q* = 0.05.

In the SHFS, after adjusting for clinical covariates, baseline LVMI, and baseline lipids, longitudinal changes in 148 lipids (52 known) were significantly associated with change in LVMI at *P* < 0.05. Of the 52 known lipids, changes in LPE(20:4), PC(35:4), and PC(37:4) were inversely associated, whereas changes in CE(22:2) and SM(d34:0) B were positively associated with change in LVMI at *q* < 0.05 (Figure 4 and Supplemental Table 3). Among lipid species associated with changes in LVMI, changes in most of them were also associated with changes in other cardiometabolic factors (e.g., insulin, insulin resistance) (Supplemental Figure 3).

### Results from sensitivity analyses in the SHFS.

Further adjustments for HDL-c, triglycerides, and the use of antihypertensive drugs slightly attenuated the associations between the identified lipids and risk of LVH, while the observed associations between lipid species and LVMI remained largely unchanged (Supplemental Tables 4 and 5). Sex-specific analysis indicated that the associations between 7 known lipids — including CER(d42:2) A, DAG(38:5), PC(17:1/22:5), PC(35:4), PC(36:6), SM(d38:1) B, and TAG(58:10) — and LVMI significantly differ in males and females (all *q* < 0.05). Results for sex-specific analyses are shown in Supplemental Table 6. Obesity status and diabetes status did not modify the association between lipid species and LVMI or the risk of LVH (Supplemental Table 7). Results from the time-varying model and the model incorporating an interaction term (lipids × time) indicated that the observed associations between lipids and LVMI remained largely unchanged (Supplemental Table 8).

## Discussion

To our knowledge, this is one of the first large-scale longitudinal lipidomic studies, comprising 1,755 unique American Indians from a community-dwelling prospective cohort, and in this study, we have several important findings. First, cross-sectional analysis reveals that altered levels of multiple individual lipid species, including glycerophospholipids, sphingolipids (e.g., SMs, CERs), glycerolipids (e.g., DAGs, TAGs), cholesterol esters, and FAs, were significantly associated with LVMI in American Indians. Some lipids were replicated in an independent cohort consisting of individuals with similar age but different racial/ethnic backgrounds. Second, our prospective association analysis demonstrated that baseline levels of 134 lipids, largely glycerophospholipids, SMs, and TAGs, were inversely associated with the risk of LVH, independent of known risk factors. Some of them were also inversely associated with incident CHD. Third, our repeated measurement analysis shows that longitudinal changes in several lipid species (e.g., PCs, SMs, CEs) were significantly associated with change in LVMI, independent of clinical factors and baseline LVMI and lipids. Together, our results demonstrate that perturbed lipid metabolism is associated with LVMI and risk of LVH in American Indians.

We found that baseline levels of multiple glycerophospholipids [e.g., PCs, PC(P)/PC(O), PE(P)/PE(O)] were inversely associated with LVMI and risk of LVH in American Indians. Longitudinal changes in glycerophospholipids were also significantly associated with changes in LVMI and cardiometabolic factors, such as BMI, blood pressure, fasting plasma glucose (FPG), and insulin resistance. The observed associations of phospholipids with LVH in our study appear to be in agreement with previous studies showing that some PCs [e.g., LPC(20:4), PC(38:4), PC(38:6)] were inversely associated with CVD mortality in other racial and ethnic groups (19–21). The inverse associations of ether-glycerophospholipids [e.g., PC(P)/PC(O), PE(P)/PE(O)] with LVMI, as well as risk of LVH and CHD observed in our study, corroborate previous studies demonstrating that baseline levels of ether-glycerophospholipids [e.g., PC(p-18:1/20:4)/PC(o-18:2/20:4), PC(p-36:2)/PC(o-36:3), PC(p-38:6), PC(p-40:6), PC(o-36:5), PE(p-18:1)] were inversely associated with risk of diabetes (22, 23), CHD (24), CVD mortality (19, 25), hypertension (26), and LV dysfunction (27) in American Indians and White participants.

PCs, the most abundant glycerophospholipids in human cardiac tissue, are key components of cell membrane and are involved in many biological processes including cell signaling, oxidative stress, and metabolism (28). PCs are the structural components of cellular membranes. The bioactive lipid products resulting from their hydrolysis, such as lysoPCs, are formed through a process facilitated by lipoprotein-associated phospholipase A2 (Lp-PLA2) (29), which plays an important role in vascular inflammation, oxidative stress, and other biological processes (29, 30). Elevated blood level of Lp-PLA2 has been linked to cardiac remodeling (31) and subsequent cardiovascular events (32), suggesting a potential connection between altered PCs, oxidative stress, inflammation, cardiac remodeling, and CVD. Ether-glycerophospholipids are a class of glycerophospholipids characterized by the presence of an ether bond at the *sn-1* position (33). They may function as potential endogenous antioxidants and are involved in various signaling pathways (33, 34). Perturbed ether-glycerophospholipid metabolism has been implicated in cardiac remodeling (35) and may also contribute to CHD by exacerbating atherosclerotic plaque formation and promoting oxidization (24, 36).

Besides glycerophospholipids, some long-chain unsaturated SMs and certain CERs were inversely associated with incident LVH in our study. In support of these findings, our group has previously reported that some SMs [e.g., SM(d36:2), SM(d40:1), SM(d40:2), SM(d41:2)], and CER(d40:2) were inversely associated with all-cause mortality (37), diabetes (22), hypertension (26), and depression (38) in the same group of American Indian participants. In addition, we found that some CERs, such as CER(d42:2) A, and CER(d40:0), were positively associated with LVMI. These findings appeared to be in agreement with previous studies reporting positive associations of CER(d42:2) and CER(d40:0) with LVMI (39, 40) and CHD (41) in White participants. Interestingly, while 1 study shows that CER(d40:2) was positively associated with CVD risk in a Chinese population (mean age, 49) over a mean follow-up of 12.9 years (42), we found an inverse relationship between CER(d40:2) [or CER(d18:2/22:0)] and the risk of LVH and CHD in American Indians. Our findings corroborate another study showing that plasma levels of CER(22:0) were inversely associated with the risk of heart failure in White participants and African Americans (43). CERs are complex bioactive lipids that play crucial roles in endothelial function, inflammation, and apoptosis (44, 45), but different species of CERs may have distinct effects. For instance, CER(16:0) increases apoptosis (46), while CER(22:0) protects against hypoxia-induced apoptosis (47). Some CERs, such as CER(d18:1/16:0), CER(d18:1/18:0) and CER(d42:2), have been widely reported to be positively associated with the risk of CHD (43, 44, 48–52). However, we did not find significant associations between CER(d18:1/16:0) and CER(d18:1/18:0) with LVMI or LVH. This discrepancy may be due to the differences in genetic makeup and/or environmental exposures (e.g., lifestyle, behavior) between American Indians and other ethnic groups (53, 54).

Some FAs [e.g., FA(18:3), FA(20:5)] were found to be inversely associated with LVMI in our study. These results appeared to be in line with previous studies showing a potential beneficial role of FAs in antiinflammation (55), blood pressure regulation (56, 57), and cardioprotection (58). FA(18:3), also known as α-linolenic acid (ALA), is a precursor to long-chain omega-3 FAs. ALA enhances membrane fluidity incorporating into PCs (59). Additionally, ALA is converted into other omega-3 FAs, such as FA(20:5), also known as eicosapentaenoic acid (EPA). EPA plays a significant role in membrane stabilization (59), antiinflammatory regulation (59), and blood pressure regulation (56). Previous epidemiological studies (56) demonstrated that omega-3 FA intake above the recommended level of 3 g/d were associated with additional benefits in lowering blood pressure among groups at high risk for CVD. Furthermore, evidence (60) shows that EPA was associated with a reduced risk of CVD mortality. On the contrary, we found that FA(22:1) was positively associated with LVMI. This finding appears to be consistent with previous evidence suggesting that high levels of FA(22:1) were associated with reduced heart function (61) and blood pressure regulation (62). Together, these findings suggest that FAs may play different roles in modulating inflammation and hypertension as well as other biological processes that are involved in cardiac remodeling and CVD.

Our repeated-measurement analysis revealed the association between longitudinal changes in plasma lipidome and change in LVMI above and over baseline measurements and clinical factors. Specifically, changes in CEs, sphingolipids (e.g., SMs, CERs), FAs, glycerophospholipids (e.g., PCs, PEs, and glycerolipids (e.g., TAGs, DAGs) were associated with changes in LVMI as well as changes in cardiovascular risk factors. Some of the identified cholesterol esters [e.g., CE(16:1), CE(22:6)] were also enriched in atherosclerotic plaques (63). Cholesterol esters are primarily synthesized in plasma through the transfer of FAs to cholesterol from PC (64). Altered FA metabolism may influence changes in LVMI by regulating the overexpression of acyl-coenzyme A synthetase-1 (ACSL1), a key enzyme in mitochondrial oxidative metabolism in the heart (65, 66). Perturbations in glycerophospholipids may indicate oxidative stress, inflammation, and endothelial cell activation in the cardiomyocyte (67, 68). Disturbance in TAG metabolism may contribute to changes in LVMI through their roles in metabolic energy or membrane formation by multiple biological processes, such as mitochondrial β-oxidation, and insulin signaling (69, 70).

In line with previous evidence for a sex difference in LVMI (71, 72) or lipid profiles (73, 74), we found that sex modifies the associations of 7 lipids, including CER(d42:2) A, DAG(38:5), PC(17:1/22:5), PC(35:4), PC(36:6), SM(d38:1) B, and TAG(58:10), with LVMI in our sample. These sex-specific effects of lipids on LVMI observed in our study align with previous studies showing the effects of sex hormones on cardiac structure and function (71, 75–77).

We found that several lipid species (e.g., glycerolipids, glycerophospholipids, and SMs) associated with LVMI were also linked to LV diastolic dysfunction parameters, such as the ratio between early and late LV filling peak velocity (E/A ratio), deceleration time, and isovolumic relaxation time (Supplemental Tables 9–11). These findings corroborate previous metabolomic studies (78, 79) showing that altered blood metabolites were associated with LV diastolic function. They also offer new insights into the role of lipid metabolism in LV structural changes and diastolic function. Moreover, given the strong relationship between cardiac remodeling, hypertension, and heart failure, our results may help enhance our understanding of the metabolic underpinnings of diastolic dysfunction and heart failure with preserved ejection fraction (HFpEF).

Several limitations of our study should be noted. First, although our study detected many lipid species, a large proportion of them are unknown compounds. Further characterization of these unknown lipids and isomers is needed in future studies. Second, all participants included in our study are American Indians who suffer high rates of obesity and diabetes. However, we adjusted for BMI and diabetes in all our analyses. In addition, given the rising trends of obesity and diabetes worldwide, our findings could likely be generalized to other racial/ethnic groups. Moreover, some of the lipids identified in American Indians were able to be replicated in an independent cohort consisting of individuals with different racial/ethnic backgrounds, suggesting the robustness of our findings. Third, although our statistical models controlled for many known risk factors, we cannot rule out potential confounding by unknown or unmeasured variables. In addition, since blood lipids and their effects on cardiometabolic risk factors may change over time, statistical models that do not take into consideration of the time-varying effects may not be appropriate. Nonetheless, to address this limitation, we employed the inverse probability of treatment weighting (IPTW) method (80, 81) to account for the time-varying effects of these variables. We also tested the interaction between time and lipids on LVMI by adding an interaction term (time [baseline versus follow-up] × lipids) to the model. These analyses show that the observed associations of lipids with LVMI remained largely unchanged. Furthermore, due to lack of cardiac MRI data in our study population, we utilized transthoracic echocardiography to measure LVM in our analysis. Future research should use MRI for more precise measurement of cardiac remodeling. Fourth, hypertension is known to affect LVM (77, 82), but this should not be an issue for our study, as we adjusted for blood pressure in all statistical analyses. Moreover, further adjustment for use of antihypertensive medications did not change our results. Fifth, although we identified multiple individual lipid species that can significantly improve the prediction of risk for LVH beyond traditional risk factors, the interpretation of these findings should be cautious due to the lack of an external cohort with similar population settings that allows us to further confirm this finding. Furthermore, although our analysis provides the direction of association between lipid levels and LVMI or risk of LVH, increases or decreases in lipid levels alone may not be able to define distinct lipid signatures. Finally, the observational nature of our study precludes any causal inference regarding the causal role of altered lipid metabolism in LVH pathogenesis.

Our study has several strengths. First, the longitudinal profiling of plasma lipidome in a large, community-based prospective cohort represents the major strength of our study. To the best of our knowledge, the current study represents the first longitudinal study examining the relationship between change in plasma lipidome and change in LVMI or risk of LVH in any racial/ethnic groups. The relative high resolution of lipidomic analysis in a large, community-based prospective cohort of American Indians is also innovative in this field. Second, our study included over 2,700 participants from 2 different populations with diverse demographics (e.g., race, age, sex, and socioeconomic status), genetic makeups, lifestyle (e.g., smoking, diet, and physical activity), and environmental exposures. Despite these variations, we were able to replicate the associations of some lipid species with LVMI, signifying the robustness of our findings. Third, among the limited metabolomic (including lipidomic) studies on LVM or LVH, most included patients with overt CVD (12, 83–85). We focused on individuals without overt CVD or LVH to minimize the effect of various risk factors. This approach helped reduce the influence of differences in cardiac structure and function between those with and without CVD. It allowed us to better examine the association between lipids and LVM or LVH. Moreover, our statistical analyses controlled for many known cardiovascular risk factors; thus, lipids identified in our study should be independent of these risk factors. Finally, we performed cross-sectional, prospective, and repeated-measurement analyses in the same group of participants, allowing us to comprehensively examine the relationship between lipid metabolism and LVM or LVH in American Indians, a traditionally understudied minority population.

In a large-scale longitudinal lipidomic profiling of LVM and LVH, we identified distinct lipidomic signatures and potentially novel lipid species associated with LVM and LVH in American Indians, independent of traditional risk factors. These findings provide insight into the role of dyslipidemia in cardiac structure remodeling and offer potential opportunities for targeting lipid metabolism in developing novel therapeutics for early prevention or intervention of cardiometabolic disorders.

## Methods

### Sex as a biological variant.

Our study included both male and female participants. The criteria for diagnosing LVH differ between sexes (86–88). Sex was defined at birth based on biological characteristics. Our findings are relevant to both sexes. Sex-specific effects of lipids on LVMI were also examined in our study.

### Study populations.

We leveraged 2 diverse populations to perform lipidomic analyses as described below. Cross-sectional, prospective, and repeated-measurement analyses were first performed in the SHFS. Putative lipids associated with LVMI were then replicated in a biracial cohort, the BHS.

The Strong Heart Study (SHS) initially focused on CVD and its risk factors in American Indians. Recognizing genetics’ role in CVD, SHS expanded into the SHFS (2001–ongoing), a family-based study aimed at identifying genetic, metabolic, and behavioral factors for CVD (4, 16, 89). Briefly, 2,786 tribal members (aged 14 years and older) residing in Arizona, North Dakota, South Dakota, and Oklahoma, USA, were recruited and examined at baseline (2001–2003) and reexamined after an over-5-year follow-up (2006–2009). Detailed descriptions of the SHFS study design, laboratory protocols, and phenotype collection have previously been described (4, 16, 89). Participants received a personal interview and a physical examination at each visit, during which fasting blood samples were collected for laboratory tests. Laboratory methods were reported previously (4). A total of 1,755 individuals (62.5% females; mean age at baseline, 39.9 years) who were free of overt CVD at baseline and had complete clinical and lipidomic data were included in the current analysis. Supplemental Figure 2 illustrates the procedures for participants’ selection and statistical analyses in the SHFS.

The BHS (1972–ongoing) is a biracial epidemiological study (35% Black, 65% White participants) designed to investigate early-life cardiometabolic risk factors in children and adolescents living in a semirural Louisiana, USA, community using serial cross-sectional surveys (17, 18). BHS participants eligible for the present study were adults (aged 34–58 years) with complete information for clinical data, including echocardiography, and metabolomic data. We excluded participants with overt CVD at the time of blood drawn for metabolomic analysis, resulting in a final sample of 973 participants (58% females; mean age, 47.9 years).

### Assessment of LVM and LVH by echocardiography.

In the SHFS, LV dimensions were measured by qualified cardiac sonographers using the Acuson Sequoia 256 Cardiac Ultrasound Machine with 2.5–3.5 MHz probe and evaluated by a single experienced cardiologist who was blinded to the individuals’ clinical features. In the BHS, 2-dimensional and tissue Doppler echocardiography were performed by trained cardiac sonographers at the BHS field office. Detailed methods for the measurement of LVM and LVH had been described previously (79, 90). Briefly, for both studies, LVM was calculated according to the Devereux formula (91) and indexed to average height in meters^2.7^ to obtain the LVMI (92). An elevated myocardial relative wall thickness (RWT) was defined as greater than 0.42 cm (93). The presence of LVH was defined as LVMI greater than 46.7 g/m^2.7^ and 49.2 g/m^2.7^ in women and men, respectively (86–88). LV geometry was considered concentric when RWT was > 0.42 cm. Four categories of LV geometry were defined: (a) normal (normal RWT and LVMI); (b) eccentric hypertrophy (normal RWT and high LVMI); (c) concentric hypertrophy (high RWT and high LVMI); and (d) concentric remodeling (high RWT and normal LVMI) (92). LV diastolic function was evaluated using the ratio of early LV filling peak velocity (E) to late LV filling peak velocity (A) (E/A ratio), deceleration time of the E wave, and isovolumic relaxation time (94).

### Assessment of clinical covariates.

In the SHFS, information for demographics, lifestyle, medical history, and use of prescription medications was collected using standard questionnaires as previously described (4, 16). Smoking status was categorized as current smokers, former smokers, and never smokers. Anthropometric measures including body height, weight, waist circumference, and fasting blood samples were obtained through physical examinations at each visit. BMI was calculated as body weight in kilograms divided by the square of height in meters. FPG, insulin, and clinical lipids, including total cholesterol, triglycerides, LDL-c, and HDL-c, were measured by standard laboratory methods (16). Hypertension was defined as blood pressure ≥ 140/90 mmHg or use of antihypertensive medications. Type 2 diabetes was defined as FPG ≥ 126 mg/dL or use of hypoglycemic drugs. Insulin resistance was assessed using homeostatic model assessment (HOMA) (22). Estimated glomerular filtration rate (eGFR) was calculated using the CKD Epidemiology Collaboration (CKD-EPI) (95). A CVD event was defined as any definite or possible fatal and nonfatal myocardial infarction, CHD, sudden cardiac death, congestive heart failure, or stroke as previously described (16). Information on the use of lipid-lowering medications and antihypertensive medications was also collected at each visit (96).

In the BHS, information on lifestyle and clinical factors, such as BMI; blood pressure; smoking; use of lipid-lowering medication; clinical lipids including total cholesterol, triglycerides, LDL-c, and HDL-c; eGFR; and definition of CVD event, were obtained using previously described methods (79, 97).

### Ascertainment of incident CHD.

In the SHFS, baseline information was collected in 2001–2003, and living participants were followed through December 31, 2020. Detailed methods for the ascertainment of incident CHD have been described previously (98, 99). Briefly, CHD included definite CHD (fatal or nonfatal), definite myocardial infarction (fatal or nonfatal), and sudden death due to CHD. CHD events were ascertained by annual review of hospitalization, death records, and self-reports (with subsequent medical record verification) during follow-up visits. Time to event was determined based on the date of baseline examination (2001–2003) to either the date of the first CHD event or the last follow-up. For participants who experienced more than 1 CHD event during the follow-up period, the earliest event date was used in the analysis. Information for incident CHD events was unavailable in the BHS.

### Lipidomic data acquisition, preprocessing, and quality control.

In the SHFS, methods for blood sample collection, lipidomic data acquisition, processing, and normalization have been described previously (22). Briefly, relative abundance of molecular lipid species in fasting plasma samples at 2 time points (~5 years apart) was quantified by untargeted liquid chromatography–MS (LC-MS). Standard methods (100) were used to quantify the lipids species. After excluding outlier and individuals with prevalent CVD or those with missing covariates, the final analysis included 1,755 participants (1,755 at baseline; 1,581 at follow-up) with complete clinical and lipidomic data, covering 1,542 lipids (518 known). No clear batches were observed in our lipidomic data (Supplemental Figure 1). Additional information on lipids assignment, internal standards, coefficient of variation, instrumental drift, and handling of missing values is described in the Supplemental Methods.

In the BHS, metabolomic profiling was performed using serum samples collected during the 2013–2016 visit by untargeted, ultra-high performance LC–tandem MS (UPLC-MS/MS). Detailed methods for blood sample collection, data acquisition, processing, and normalization of the metabolomic data have been described previously (79). Of the 152 lipids identified in the SHFS (*P* < 0.05), 21 lipids were also available in the BHS, and we used them to replicate our findings in the SHFS (Supplemental Methods). After further excluding individuals with prevalent CVD or those with missing covariates, a total of 973 BHS participants (58% females, 35% Black, 65% White) with available metabolomic data were included in the replication analysis.

### Cross-sectional association analysis.

To identify lipid species associated with LVMI, we constructed generalized estimating equation (GEE) models in the SHFS using samples collected at baseline (*n* = 1,755) and follow-up (*n* =1,581), separately. Results at both time points were then combined by fixed-effects meta-analysis. The models adjusted for age, sex, study center, BMI, smoking status (current smoker versus ever smoker versus nonsmoker), SBP, diabetes, eGFR, LDL-c, and the use of lipid-lowering medication at the time blood samples were drawn. The GEE model was used here to account for the relatedness among family members. The putative lipids (raw *P* < 0.05) in the SHFS meta-analysis were then validated in the BHS (*n* = 973, external replication) using linear regression, adjusting for age, race (White versus Black), sex, BMI, smoking status (current smoker versus ever smoker versus nonsmoker), SBP, diabetes, eGFR, LDL-c, and the use of lipid-lowering medication. Replication was defined as lipids with *q* < 0.05 and consistent directions of association across both cohorts. Meta-analysis was performed by inverse-variance weighted random-effects model to combine results across the 2 cohorts.

### Prospective association analysis.

To identify baseline plasma lipids associated with risk of LVH, we constructed logistic GEE models. In this model, baseline lipid was the predictor, and the status of incident LVH (yes/no) was the outcome, adjusting for age, sex, study center, BMI, smoking status (current smoker versus ever smoker versus nonsmoker), SBP, diabetes, eGFR, LDL-c, and the use of lipid-lowering medication at baseline. Participants with prevalent LVH at baseline were excluded from this analysis.

To assess whether the identified lipids improve the prediction of LVH risk beyond known clinical factors, we used data from 2 study centers (North/South Dakota and Arizona) as the training set (*n* = 762, 42 cases) and those from another study center (Oklahoma) (*n* = 675, 37 cases) as the testing set. We then compared a base model including traditional risk factors only (age, sex, BMI, smoking status [current smoker versus ever smoker versus nonsmoker], SBP, diabetes, eGFR, LDL-c, and the use of lipid-lowering medication) and a model containing both traditional risk factors and the significant lipids identified in the prospective analysis. The incremental predictive value of lipids over known risk factors was assessed by area under the receiver operating characteristic curve (AUROC) (101).

To further examine whether the identified LVH-related plasma lipids are associated with incident CHD after 18-year follow-up, we constructed frailty Cox proportional hazards models. In this model, baseline level of the identified LVH-related lipid was the predictor, and the time to event was the outcome, adjusting for age, sex, study center, BMI, smoking status (current smoker versus ever smoker versus nonsmoker), hypertension, diabetes, LDL-c, HDL-c, and eGFR at baseline. The frailty term was used here to account for the relatedness among family members.

### Repeated measurement analysis.

Of 1,148 participants free of overt CVD and prevalent LVH at baseline and follow-up, we constructed linear GEE models to examine the longitudinal association between change in lipid species and change in LVMI between baseline and 5-year follow-up. In the model, change in LVMI was the outcome, and change in the relative abundance of each individual lipid was the predictor. The model adjusted for age, sex, study center, smoking status (current smoker versus ever smoker versus nonsmoker), diabetes, use of lipid-lowering medication, and changes in BMI, SBP, eGFR, LDL-c, baseline LVMI, and lipids. Lipids with *q* < 0.05 were considered statistically significant. The associations between changes in lipids and changes in cardiometabolic factors including BMI, SBP, diastolic blood pressure, FPG, insulin, and insulin resistance were similarly examined.

Of note, due to the lack of information on incident CHD and longitudinal lipidomics data in the BHS, the above-described prospective association analysis and repeated measurement analysis were only conducted in the SHFS.

### Sensitivity analysis.

To evaluate the robustness of our results, we conducted the following sensitivity analyses. First, to examine the potential effect of bulk lipids (e.g., HDL-c, triglycerides) and the use of antihypertensive medications (yes/no) on our results, we additionally adjusted for these variables in the statistical models. Second, to examine whether sex, obesity status, or diabetes status modulates the association between lipid species and LVMI or the risk of LVH, we further included an interaction term (sex [male versus female] × lipids, or obesity status [yes/no] × lipids, or diabetes status [yes/no] × lipids) in the statistical model. Third, to examine how time and the progression of cardiometabolic risk factors may affect the association between lipids and LVMI, we employed the IPTW method (80, 81) to account for the effects of time-varying covariates (e.g., BMI, smoking, SBP, diabetes, eGFR). Additionally, we tested the interaction between time and lipids on LVMI by adding an interaction term between time (baseline versus follow-up) and lipids to the statistical model.

### Statistics.

All continuous variables including level of lipids were standardized to zero mean and unit variance. *P* values less than 0.05 were deemed significant. Multiple testing was controlled by FDR using the Storey’s *q* value method (102, 103). Statistical analysis was conducted using R Studio (version 9).

### Study approval.

All SHFS and BHS participants provided informed consent. The SHFS protocols were approved by the IRBs of the participating institutions and the American Indian tribes. The BHS was approved by the IRB of the Tulane University Health Sciences Center.

### Data availability.

The SHFS phenotype data used in this study can be requested through the Strong Heart Study Coordinator Center (https://strongheartstudy.org/). The SHFS lipidomic data can be obtained from the corresponding author upon a reasonable request. Clinical and metabolomic data in the BHS can be requested via https://bogalusaheartstudy.org/ All codes used for the statistical analyses are available on GitHub at https://github.com/stephanieArtero/LVM/commit/fcb753b Values for all data points in graphs are reported in the Supporting Data Values file.

## Author contributions

JZ conceptualized and designed the study, obtained the funding and generated the data. MC and ZH conducted the statistical analyses. MC drafted the manuscript. GM, JR, JL, and JZ helped with data interpretation and contributed to drafting of the manuscript. OF collected the LC-MS data and contributed to data interpretation. MJR, RBD, RRF, YZ, JGU, SAC, and TNK provided critical review and contributed to discussion of the manuscript.

## Supplementary Material

Supplemental data

ICMJE disclosure forms

Supplemental tables 1-11

Supporting data values

## Figures and Tables

**Table 1:**
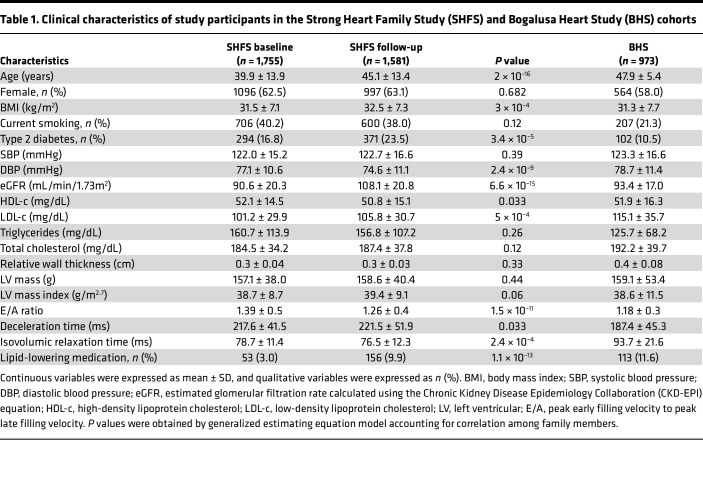
Clinical characteristics of study participants in the Strong Heart Family Study (SHFS) and Bogalusa Heart Study (BHS) cohorts

## Version 1 10/15/2024

In-Press Preview

## Version 2 11/22/2024

Electronic publication
