# Supplementary material for: Longitudinal lipidomic profiles of left ventricular mass and left ventricular hypertrophy in American Indians
Source: JCI Insight. 2024 Oct 15;9(22):e181172. doi: 10.1172/jci.insight.181172 (PMC11601894; doi:10.1172/jci.insight.181172)

Longitudinal lipidomic profiles of left ventricular mass and left ventricular hypertrophy in American Indians

Mingjing Chen,[1] Zhijie Huang,[2] Guanhong Miao,[1] Jin Ren,[1] Jinling Liu,[1] Mary J. Roman,[3] Richard B. Devereux,[3] Richard R. Fabsitz,[4] Ying Zhang,[5] Jason G. Umans,[6] Shelley A. Cole,[7] Tanika N. Kelly,[8] Oliver Fiehn,[9] Jinying Zhao[1]

[1]Department of Epidemiology, College of Public Health & Health Professions and College of Medicine, University of Florida, Gainesville, FL

[2]Department of Epidemiology, Tulane University School of Public Health and Tropical Medicine, New Orleans, LA

[3]Division of Cardiology, Weill Cornell Medical College, New York, NY

[4]Missouri Breaks Industries Research Inc, Eagle Butte, SD

[5]Department of Biostatistics and Epidemiology, University of Oklahoma Health Sciences Center, Oklahoma City, OK

[6]MedStar Health Research Institute, Hyattsville, MD; and Georgetown-Howard Universities Center for Clinical and Translational Science, Washington, DC

[7]Texas Biomedical Research Institute, San Antonio, TX

[8]Department of Medicine, University of Illinois Chicago, Chicago, IL

[9]West Coast Metabolomics Center, University of California-Davis, CA

Correspondence to: Jinying Zhao, MD, PhD
Professor, Department of Epidemiology
Director, Center for Genetic Epidemiology and Bioinformatics
College of Public Health and Health Professions and College of Medicine, University of Florida
2004 Mowry Rd, Gainesville, FL 32610
Tel: 352-273-5933; Fax: 352-273-5365
Email: jzhao66@ufl.edu

Supplemental Figures: 3

Supplementary Methods

Lipidomic data acquisition, pre-processing and quality control. Methods for blood sample collection, lipidomic data acquisition, processing, and normalization int the Strong Heart Family Study have been described previously.[1] Briefly, plasma samples were first extracted based on a modified liquid-liquid extraction method (cold methanol/ MTBE/water). The extracted samples were then subjected to lipidomics analysis by LC-MS in both positive and negative ionization modes.

The LC–MS systems used, and injection volumes are given below:

LC system	MS system	R (fwhm)	resuspension volume (μL)	injection volume (μL)
Agilent 1290 Infinity	Agilent 6530	10,000	200	1.5
Agilent 1290 Infinity	Agilent 6550 iFunnel	20,000	500	1.5

Each LC system consisted of a pump, a column oven, and an autosampler. Lipids were separated on an Acquity UPLC CSH C18 column (100 × 2.1 mm; 1.7 μm) coupled to an Acquity UPLC CSH C18 VanGuard precolumn (5 × 2.1 mm; 1.7 μm) (Waters, Milford, MA). The column was maintained at 65 °C at a flow-rate of 0.6 mL/min. The mobile phases consisted of (A) 60:40 (v/v) acetonitrile: water with ammonium formate (10 mM) and formic acid (0.1%) and (B) 90:10 (v/v) isopropanol: acetonitrile with ammonium formate (10 mM) and formic acid (0.1%). The separation was conducted under the following gradient: 0 min 15% (B); 0–2 min 30% (B); 2–2.5 min 48% (B); 2.5–11 min 82% (B); 11–11.5 min 99% (B); 11.5–12 min 99% (B); 12–12.1 min 15% (B); and 12.1–15 min 15% (B). Sample temperature was maintained at 4 °C. Detailed instrumental parameters are described here:

Agilent 6530 QTOF MS

Analyses on both quadrupole/time-of-flight mass spectrometers Agilent 6530 with a Dual Spray ESI ion source (Agilent Technologies, Santa Clara, CA) were performed at the high sensitivity mode. Simultaneous MS1 and MS/MS (All Ion MS/MS) acquisition was used. The parameters were ESI polarity, positive; capillary voltage, 3.5 kV; nozzle voltage, 1 kV; gas temperature, 325 °C; drying gas (nitrogen), 8 L/min; nebulizer gas (nitrogen), 35 psi; sheath gas temperature, 350 °C; sheath gas flow (nitrogen), 11 L/min; MS1 acquisition speed, 2 spectra/s; MS1 mass range, m/z 60–1700; MS/MS acquisition speed, 2 spectra/s; MS/MS mass range, m/z 60–1700; collision energy, 25 eV. The instrument was tuned using an Agilent tune mix. A reference solution (m/z 121.0509, m/z 922.0098) was used to correct small mass drifts during the acquisition.

Agilent 6550 iFunnel QTOF MS

Analyses on an Agilent 6550 iFunnel QTOF with a Dual Spray ESI ion source (Agilent Technologies) were performed at the high sensitivity mode. Simultaneous MS1 and

MS/MS (All Ion MS/MS) acquisition was used. The parameters were ESI polarity, positive; capillary voltage, 3.5 kV; nozzle voltage, 1 kV; gas temperature, 200 °C; drying gas (nitrogen), 14 L/min; nebulizer gas (nitrogen), 35 psi; sheath gas temperature, 350 °C; sheath gas flow (nitrogen), 11 L/min; MS1 acquisition speed, 2 spectra/s; MS1 mass range, m/z 60–1700; MS/MS acquisition speed, 2 spectra/s; MS/MS mass range, m/z 60–1700; collision energy, 25 eV. The instrument was tuned using an Agilent tune mix. A reference solution (m/z 121.0509, m/z 922.0098) was used to correct small mass drifts during the acquisition.

Internal standards

The following internal standards were used for both correction of small metabolite drifts and as surrogates for quantifications:

Concentrations (nmol/mL) of the Internal Standards Spiked in Blood Plasma internal standard *a*	concentration (nmol/mL plasma)	used for retention time correction	used for quantification
CUDA	150b	✓	×
Sphingosine (d17:1)	17.9	✓	×
LPE(17:1)	49.3	✓	✓
LPC(17:0)	30	✓	✓
MG(17:0/0:0/0:0)	178	✓	×
DG(18:1/2:0/0:0)	230	✓	×
PC(12:0/13:0)	1.44	✓	✓
DG(12:0/12:0/0:0)	101	✓	✓
d7-Cholesterol	155	✓	✓
SM(d18:1/17:0)	8.54	✓	✓
PG(17:0/17:0)	57.7	✓	×
Cer(d18:1/17:0)	13.9	✓	✓
PE(17:0/17:0)	16	✓	✓
*d*5-TG(17:0/17:1/17:0)	6.74	✓	✓
CE(22:1)	771	✓	✓

Overall precision achieved was analyzed by plasma pool quality controls, obtained from Bioreclamation (now BioIVT) company, with the following coefficient of variance:

	biorec %RSD	NIST %RSD
Internal standards	3.3%	10.1%
Known compounds	4.3%	12.6%
Unknown compounds	12.8%	19.6%

<u>No drift was observed</u>. A total of 207 Bioreclamation pool plasma quality controls were analyzed, along with a total of 54 NIST SRM1950 pool plasma quality controls.

Quality Control

Quality control was assured by (i) randomization of the sequence, (ii) injection of 10 pool samples to equilibrate the LC–MS system before actual sequence of samples; (iii) injection of pool samples at the beginning and the end of the sequence and between each 10 actual samples, (iv) injection of NIST SRM 1950 at the beginning of the sequence and after injection of 100 actual samples; (v) procedure blank analysis, (vi) checking the peak shape and the intensity of spiked internal standards and the internal standard added prior to injection, and (vii) monitoring mass accuracy of internal standards during the run.

Data Processing

Raw data files were converted to ABF format using Reifycs Abf (Analysis Base File) Converter (accessible at: http://www.reifycs.com/AbfConverter/). For data processing, MS-DIAL (v. 2.52) software[2] program was used. The following parameters were used: retention time begin, 0.3 min; retention time end, 12.6 min; mass range begin, 280 Da; mass range end, 1500 Da; MS1 (centroiding) tolerance, 0.01 Da; smoothing level, 3 scans; minimum peak height, 500 amplitude (QTOFs), 300 amplitude (TOF), 100000 amplitude (Q Exactive HF), 1 amplitude (X500R); mass slice width, 0.05 Da; retention time tolerance for retention time–m/z (t_R–m/z) library, 0.15 min; accurate mass tolerance, 0.03 Da; retention time tolerance for alignment, 0.1 min; MS1 tolerance for alignment, 0.025 Da.

Filtering aligned peaks. The filtering process involved several steps. First, if all peak intensities across samples for a given alignment ID were missing or undetected, that alignment was removed. Second, a user-defined peak count filter was applied, where alignment IDs with a percentage of filled peaks below the threshold (default is 0%) were excluded to ensure alignments with insufficient detected peaks were not included in the analysis. Additionally, an optional step removed alignment IDs where all quality control (QC) samples had missing values, enhancing the reliability of the data by ensuring that QC samples provided meaningful intensity information.

Interpolating missing values. For alignment IDs that passed the filtering process but still contained missing values, MS-DIAL applied an interpolation method. First, the software calculated the average retention time and average m/z (mass-to-charge ratio) from the "filled" peaks, providing a reference point based on detected signals. Next, the software scanned within a defined range around the average retention time and m/z to identify the local maximum intensity value, which was then used to fill in the missing values. This process ensured that missing values were estimated based on the nearest available data points.

Lipid identification. Accurate mass and MS/MS matching was used with the public LipidBlast library of over 200,000 MS/MS spectra. Lipids were annotated covering many lipid classes and various molecular species: AC, CE, cholesterol, Cer (Cer, HexCer, Hex2Cer), DG, LPC, LPE, PC (PC, pPC/oPC), PE (PE, pPE/oPE), SM, and TG. Quantification was performed by combining data for different detected molecular species for each particular lipid (e.g., sum of [M + NH4]+, [M + Na]+, [M + K]+

adducts for each TG species) followed by normalization using (i) class-specific internal standards or (ii) sum of all annotated lipids (total ion chromatogram, TIC). For DG species, DG 12:0/12:0/0:0 was used for quantification because of its elution proximity (tR ~ 4.3 min) with all DG species (tR = 5.2–8.2 min) compared to DG 18:1/2:0/0:0 (tR ~ 3.2 min). All internal standards, including DG(18:1/2:0/0:0), MG(17:0/0:0/0:0), PG(17:0/17:0), sphingosine d17:1, and CUDA were used for retention time correction for the tR –m/z lipid library.

List of overlapping lipids between SHFS (discovery) and BHS (replication)

A list of 21 lipids available in both SHFS and BHS

lipids in the SHFS	Lipids in BHS	HMDB	Class
Cholesterol	Cholesterol	HMDB00067	Cholesterol
LPC(16:1)	2-palmitoleoyl-GPC (16:1)*	HMDB10383	Phosphocholines
LPC(20:4) A	1-arachidonoyl-GPC (20:4n6)	HMDB10395	Phosphocholines
LPC(20:4) B	1-arachidonoyl-GPC (20:4n6)	HMDB10395	Phosphocholines
AC(18:0)	Stearoylcarnitine (C18)	HMDB00848	Acylcarnitines
FA(22:1)	Erucate (22:1n9)	HMDB02068	Fatty acids
SM(d32:1) B	Sphingomyelin (d18:1/14:0, d16:1/16:0)	HMDB12097	Sphingomyelins
SM(d32:1) A	Sphingomyelin (d18:1/14:0, d16:1/16:0)	HMDB12097	Sphingomyelins
SM(d36:2) B	Sphingomyelin (d18:1/18:1, d18:2/18:0)	HMDB12101	Sphingomyelins
SM(d36:2) A	Sphingomyelin (d18:1/18:1, d18:2/18:0)	HMDB12101	Sphingomyelins
SM(d38:1) A	Sphingomyelin (d18:1/20:0, d16:1/22:0)	HMDB12102	Sphingomyelins
SM(d38:1) B	Sphingomyelin (d18:1/20:0, d16:1/22:0)	HMDB12102	Sphingomyelins
SM(d42:2) A	Sphingomyelin (d18:1/24:1, d18:2/24:0)	HMDB12107	Sphingomyelins
LPC(18:1)	1-oleoyl-GPC (18:1)	HMDB02815	Phosphocholines
CER(d34:1)	N-palmitoyl-sphingosine (d18:1/16:0)	HMDB04949	Ceramides
AC(26:0)	Cerotoylcarnitine (C26)	HMDB06347	Acylcarnitines
PC(34:4)	1-myristoyl-2-arachidonoyl-GPC (14:0/20:4)	HMDB07883	Phosphocholines
PC(38:6) C	1-palmitoyl-2-docosahexaenoyl-GPC (16:0/22:6)	HMDB07991	Phosphocholines

PC(38:6) B	1-palmitoyl-2-docosahexaenoyl-GPC (16:0/22:6)	HMDB07991	Phosphocholines
PC(38:6) A	1-palmitoyl-2-docosahexaenoyl-GPC (16:0/22:6)	HMDB07991	Phosphocholines
PC(40:6) B	1-stearoyl-2-docosahexaenoyl-GPC (18:0/22:6)	HMDB08057	Phosphocholines

Reference

1. Cajka T, Smilowitz JT, Fiehn O. Validating Quantitative Untargeted Lipidomics Across Nine Liquid Chromatography-High-Resolution Mass Spectrometry Platforms. *Analytical chemistry*. 2017;89:12360-12368. doi: 10.1021/acs.analchem.7b03404
2. Tsugawa H, Cajka T, Kind T, Ma Y, Higgins B, Ikeda K, Kanazawa M, VanderGheynst J, Fiehn O, Arita M. MS-DIAL: data-independent MS/MS deconvolution for comprehensive metabolome analysis. *Nature methods*. 2015;12:523-526. doi: 10.1038/nmeth.3393

Figure S1. Principal component analysis shows that there are no clear batches in our lipidomic data. Each dot represents a lipid measured at baseline (red) or average 5-year follow-up (green). No clear batch was observed for lipids measured at baseline and follow-up. PC1: principal component 1, PC2: principal component 2.

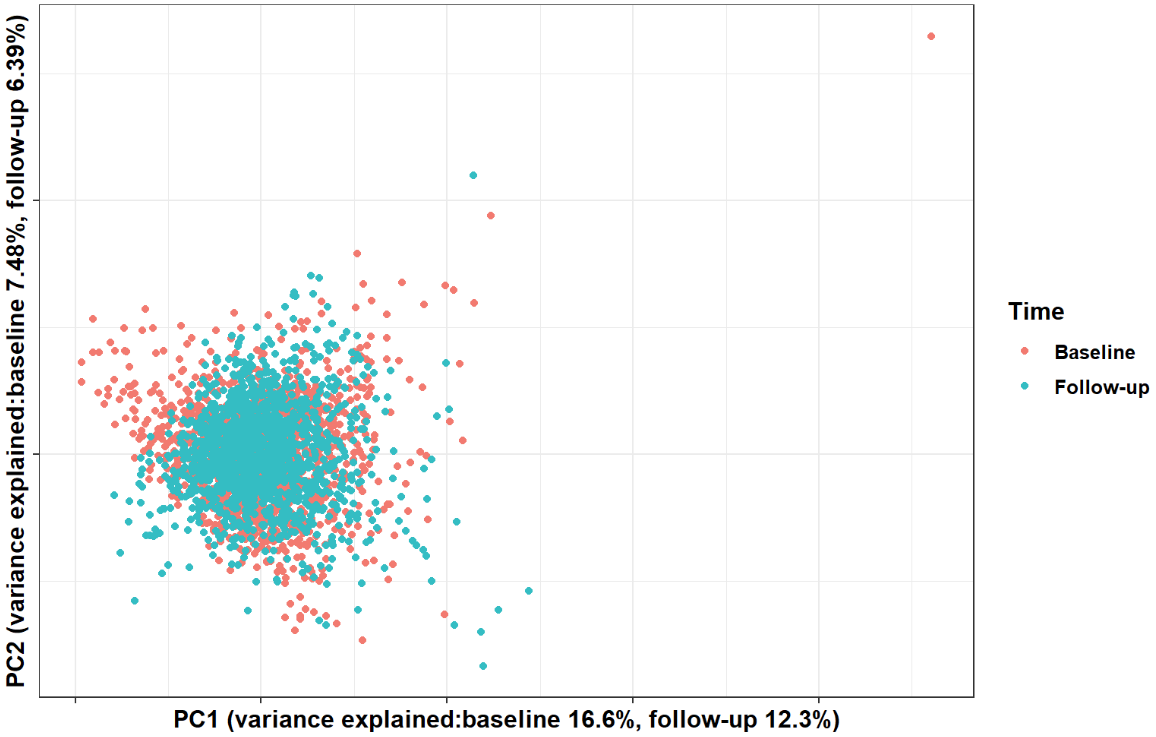

Figure S2. Flowchart describing the procedures for participants' selection and statistical analyses in the SHFS. To identify lipids associated with left ventricular mass index (LVMI), we constructed generalized estimating equation (GEE) models using the samples collected at baseline (n=1,755) and follow-up (n=1,581) in the Strong Heart Family Study (SHFS), separately. Results at both time points were then combined by fixed-effects meta-analysis. The models adjusted for age, sex, study center, BMI, smoking status, systolic blood pressure (SBP), diabetes, eGFR, LDL-c, and the use of lipid-lowering medication at the time blood samples were drawn. The putative lipids (raw $P<0.05$) in the SHFS were then validated in the Bogalusa Heart Study (BHS) (n=973, external replication) using linear regression, adjusting for age, race, sex, BMI, smoking, SBP, diabetes, eGFR, LDL-c and the use of lipid-lowering medication. To identify baseline lipid species associated with risk of left ventricular hypertrophy (LVH) in the SHFS, we conducted GEE models, adjusting for same covariates at baseline. To further assess the identified LVH-related plasma lipids predictive of incident coronary heart disease at the end of 18-year follow-up beyond clinical factors in the SHFS, we constructed frailty Cox proportional hazards model with the frailty term accounting for the relatedness among family members, adjusting for age, sex, BMI, smoking, hypertension, diabetes, LDL-c, HDL-c, and eGFR at baseline. In addition, we performed repeated measurement analysis to dissect longitudinal changes in lipids associated with changes in LVMI, adjusting for same covariates plus baseline lipids and LVMI.

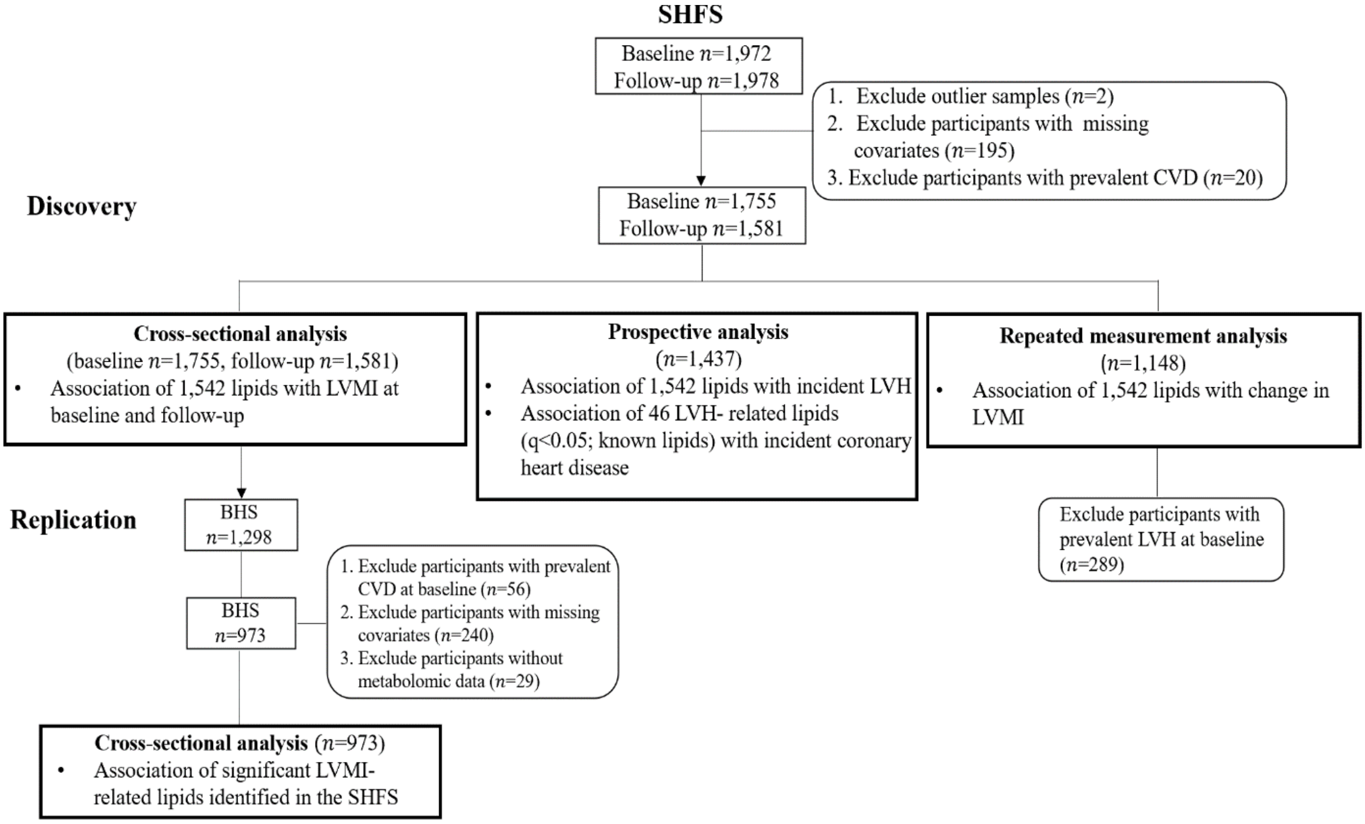

Figure S3. Heatmap showing the longitudinal associations between changes in LVMI-related lipids and changes in cardiovascular risk factors including BMI, systolic blood pressure (SBP), diastolic blood pressure (DBP), fasting plasma glucose, insulin and insulin resistance (IR) between baseline and follow-up (mean ~5-year follow-up). Each row represents a lipid, and each column represents a continuous trait. Color codes are based on regression coefficients obtained from the GEE model, in which change in lipid was the independent variable, and change in one of the traits was the dependent variable, adjusting age, sex, study center, smoking, and diabetes at baseline as well as baseline levels of the specific lipid and the cardiovascular risk factor under investigation. *P < 0.05; **q < 0.05

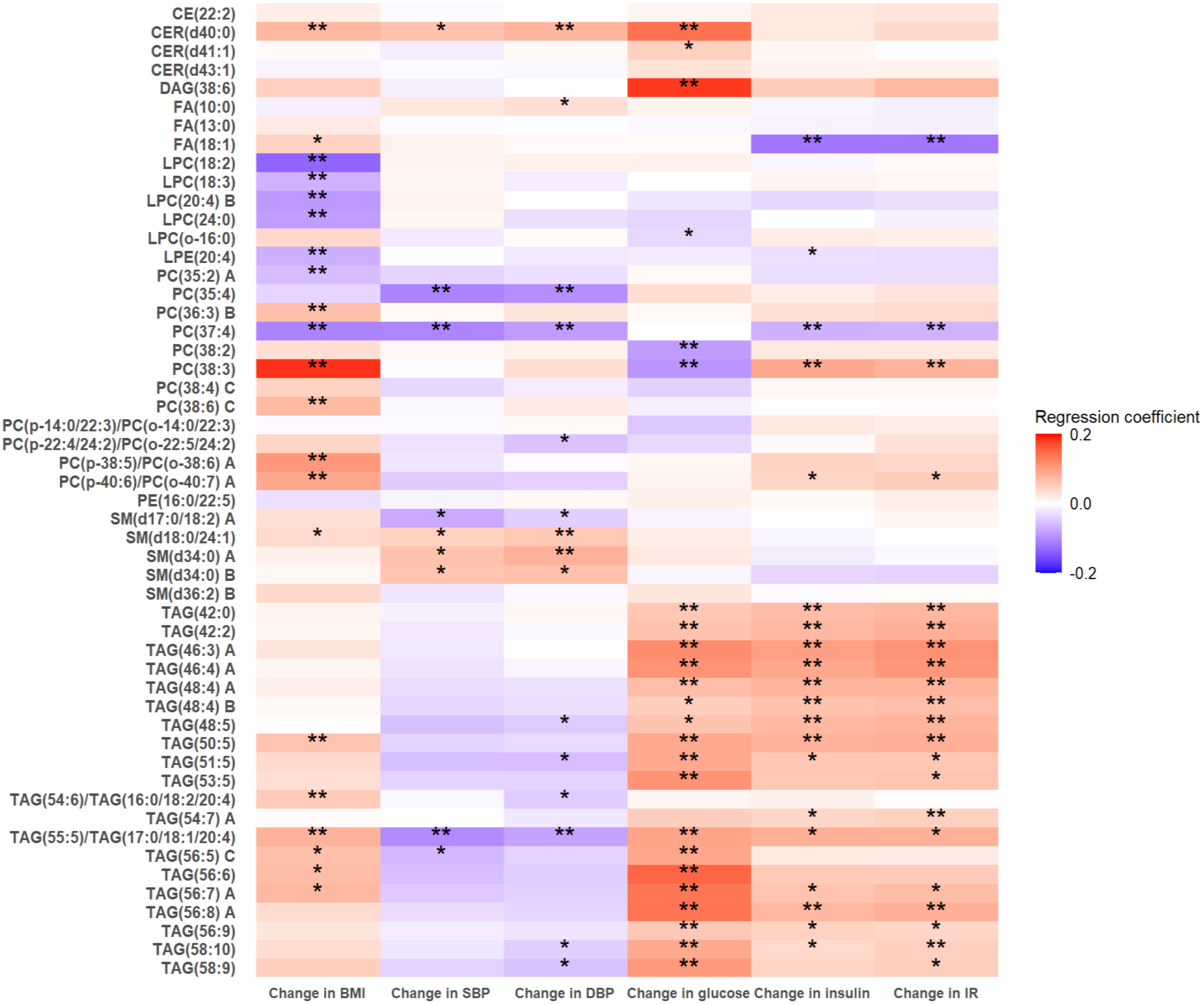

Supplement: Supplemental data [file jciinsight-9-181172-s281.pdf]
